# Investigation of Complement Component *C4* Copy Number Variation in Human Longevity

**DOI:** 10.1371/journal.pone.0086188

**Published:** 2014-01-22

**Authors:** Friederike Flachsbart, Amke Caliebe, Femke-Anouska Heinsen, Tom Hemming-Karlsen, Stefan Schreiber, Andre Franke, Almut Nebel

**Affiliations:** 1 Institute of Clinical Molecular Biology, Christian-Albrechts-University of Kiel, Kiel, Germany; 2 Institute of Medical Informatics and Statistics, Christian-Albrechts-University of Kiel, Kiel, Germany; 3 Norwegian PSC Research Center, Division of Cancer, Surgery and Transplantation, Oslo University Hospital, Rikshospitalet, Oslo, Norway; 4 Research Institute for Internal Medicine, Oslo University Hospital, Rikshospitalet, Oslo, Norway; 5 Section of Gastroenterology, Department of Transplantation Medicine, Division of Cancer, Surgery and Transplantation, Oslo University Hospital, Rikshospitalet, Oslo, Norway; 6 Division of Gastroenterology, Institute of Medicine, University of Bergen, Bergen, Norway; 7 Department of General Medicine, University Hospital Schleswig-Holstein, Kiel, Germany; 8 Popgen Biobank, Christian-Albrechts-University, Kiel, Germany; Yale School of Public Health, United States of America

## Abstract

Genetic factors have been estimated to account for about 25% of the variation in an adult's life span. The complement component C4 with the isotypes C4A and C4B is an effector protein of the immune system, and differences in the overall *C4* copy number or gene size (long *C4L*; short *C4S*) may influence the strength of the immune response and disease susceptibilities. Previously, an association between *C4B* copy number and life span was reported for Hungarians and Icelanders, where the C4B*Q0 genotype, which is defined by *C4B* gene deficiency, showed a decrease in frequency with age. Additionally, one of the studies indicated that a low *C4B* copy number might be a genetic trait that is manifested only in the presence of the environmental risk factor “smoking”. These observations prompted us to investigate the role of the *C4* alleles in our large German longevity sample (∼700 cases; 94–110 years and ∼900 younger controls). No significant differences in the number of *C4A*, *C4B* and *C4S* were detected. Besides, the C4B*Q0 carrier state did not decrease with age, irrespective of smoking as an interacting variable. However, for C4L*Q0 a significantly different carrier frequency was observed in the cases compared with controls (cases: 5.08%; controls: 9.12%; p = 0.003). In a replication sample of 714 German cases (91–108 years) and 890 controls this result was not replicated (p = 0.14) although a similar trend of decreased C4L*Q0 carrier frequency in cases was visible (cases: 7.84%; controls: 10.00%).

## Introduction

Human longevity is considered a multi-factorial phenotype, and genetic factors have been estimated to account for about 25% of the variation in adult life span [1], [2], [3], [4], [5]. Nonagenarians and centenarians have outlived the vast majority of their peers by many decades. Most of them have spent their life in good health and often markedly delay or even escape major age-related diseases [6], [7]. It has been postulated that these long-lived individuals (LLI) carry a reduced number of risk alleles for age-related diseases [8], [9]. A case in point is the apolipoprotein E*ε4 allele (risk factor for Alzheimer's and coronary heart disease) that is significantly depleted in LLI [1].

Complement C4 is a central component of the mannose-binding lectin activation pathways of the complement system that are main effectors of the adaptive and innate immune responses [10], [11], [12]. The C4 protein exists as two isotypes, the acidic C4A and basic C4B form. They are encoded by the *C4A* and *C4B* genes located in the major histocompatibility complex (MHC) class III region. *C4A* and *C4B* differ from each other only at five single nucleotide polymorphisms (SNPs) [13], [14]. Although the resulting isotypes are antigenetically quite similar (>99% amino acid sequence identity), they exhibit functional differences, for instance in chemical reactivities to substrates [15], [16], [17], [18], [19]. Furthermore, it is thought that the C4A protein is more important in immunoclearance, whereas C4B is more relevant in the defense against microbes [17].

Both *C4A* and *C4B* genes are found with varying copy numbers (CNVs), as indicated by the Database of Genomic Variants (http://projects.tcag.ca/variation) [15]. The total number of *C4A* and *C4B* genes can range between two and eight (considering homologous chromosomes). While most healthy subjects carry two *C4A* and two *C4B* genes each [15], [16], [17], heterozygous deficiency of *C4A* or *C4B* has been described in up to 30% of populations of European descent [20]. *C4B* gene deficiency (zero or one *C4B* gene in the diploid genome) is traditionally called C4B*Q0 and the corresponding situation for *C4A* is denoted as C4A*Q0 [12], [15]. In addition, both *C4* genes differ in size; they can be short (*C4S*) or long (*C4L;* due to the insertion of 6.4 kb from the endogenous retrovirus HERV-K) [21]. In Caucasians about 76% of the *C4* genes belong to the long form and 24% to the short form [18], [20]. *C4L/C4S* gene deficiency is called C4S*Q0 or C4L*Q0, respectively. The copy number and the size of *C4* genes strongly determine the plasma C4 protein concentrations and linear correlations between total *C4*, *C4A* and *C4B* gene copy number with their corresponding plasma protein concentration were observed [22]. For instance, individuals containing one or more short *C4* genes have consistently higher serum total C4 concentrations than those with long *C4* genes only, suggesting a negative epistatic effect of HERV-K retrovirus on the expression of C4 proteins [18].

The frequent variations in *C4* size and numbers render the gene an interesting marker for major histocompatibility complex disease associations [23], [24], [25], [26]. Expression and overall gene copy numbers of total *C4, C4A*, *C4B* or the *C4* gene size (*C4S* or *C4L*) may influence the strengths of innate or adaptive immunity and disease susceptibilities [15], [16], [18], [27], [28], [29]. A low *C4B* copy number has been shown to be a risk factor for cardiovascular diseases [15], [30], [31], [32], [33], [34], [35]. Furthermore, a study by Kramer et al. revealed that a low *C4B* allele frequency is associated with a shortened life span. The authors observed a significant decrease in C4B*Q0 alleles among healthy old Hungarians (>62 years) compared to a younger (<53 years) control group (from 18% allele frequency in the young, which equals 36% carrier frequency; to 5% allele frequency in the elderly, corresponding to 10% carrier frequency) [36]. In a follow-up study in 1991, Kramer et al. supported their previous findings. They detected a marked decrease in the C4B*Q0 allele frequency (p<0.0001) in a second sample of old Hungarian study participants (>60 years: 5%; equals 10% carrier frequency) compared to younger individuals (<45 years: 16%; equals 32% carrier frequency). To test if this observation was population-specific for the two investigated Hungarian samples, a second collaborative experiment in 381 healthy Icelandic people, including 73 healthy elderly individuals (>59 years) was performed. In this independent replication sample, a significant decrease in the C4B*Q0 carrier frequency was found in the elderly group from 24% to 5% [37]. In a subsequent Icelandic follow-up study, however, no statistically significant age-associated decrease was detected for the C4B*Q0 carrier frequency [30]. Because smoking severely affects health and survival on a global scale [38], [39], the authors tested whether the increased risk of early morbidity observed in carriers of C4B*Q0 could be associated with smoking as an interacting variable. After dividing the whole Icelandic sample into smokers and non-smokers, a significant decrease in C4B*Q0 carrier frequency with age (17–39 years: 19%, 40–49 years: 20%, 50–59 years: 4%, 60–93 years: 0%; χ^2^ test for trend; p = 0.0079) was seen only among smokers, indicating that *C4B* low copy number might be a genetic trait that is manifested only in the presence of the environmental risk factor “smoking” [30]. All data on *C4B* copy number variation in Hungarians and Icelanders are summarized in Szilagyi et al. 2008 [15]. In an Italian study sample of 77 centenarians (100–107 years), 89 elderly subjects (70–89 years) and 235 young subjects (18–49 years) the negative influence of C4B*Q0 alleles on life span was not confirmed [40].

Here, we employed our large study sample of German individuals to *i)* investigate the role of *C4* copy number variation in human longevity, to *ii)* potentially validate the association of C4B*Q0 carrier state with life span and to *iii)* test the hypothesis of “smoking” as an effecting variable. About 700 LLI between 94 and 110 years of age and approximately 900 younger individuals (19–75 years) have been analyzed for the *C4A, C4B, C4S* and *C4L* allele distribution. To evaluate whether differences in C4B*Q0 carrier frequency could be detected in smokers, we additionally performed a stratified analysis for smokers and non-smokers.

## Materials and Methods

### Study participants

In this study, we investigated ∼700 LLI (male/female ratio approximately 1/1; age-range 94–110 years) and ∼900 younger controls (male/female ratio approximately 1/1; age-range 19–75 years) drawn from German population-based collections [41], [42] for *C4* copy number variation. The proportion of recent smokers was a little above 3% for LLI and a little below 40% for controls. A detailed description of the samples and the recruitment procedure is given elsewhere [43] and in Supplementary Table S1a and S1b in File S1.

The sample used to investigate the *C4L* alleles in the replication experiment comprised 714 LLI (male/female ratio approximately 1/10; age-range 91–108 years) and 890 controls (male/female ratio approximately 1/10; age-range 18–79), for details see Supplementary Table S1a and S1b in File S1.

All participants gave written informed consent to participate within the study. Approval for the project was obtained by the Ethics Committee of the Medical Faculty of the Christian-Albrechts-University, Kiel.

### Genotyping

Genotyping of *C4* copy numbers (determination of *C4A*, *C4B*, *C4L*, and *C4S* dosage) was performed using the TaqMan chemistry-based real-time PCR technique (*C4A*, Hs07226349; *C4B*, Hs07226350; *C4L*, Hs07226352; *C4S*, Hs07226351) (Life Technologies Corporation, Foster City, CA) [16]. For each sample four replicates were genotyped and samples with data for fewer than three replicates were excluded from further analyses. All copy number data are listed in Data S1.

### Statistical analyses

The copy number calculation was performed using the CopyCaller^TM^ Software (Life Technologies Corporation, Foster City, CA). The copy number assignment was cross-checked internally for each individual as the sum of *C4A* copies plus *C4B* copies should equal the sum of *C4L* copies plus *C4S* copies. If the equality was violated, the corresponding individual was excluded from the analyses. Further quality control for each CNV calling included: *a)* removal of all samples with analysed replicate number 0, 1 or 2; *b)* removal of all samples with confidence <0.95 and |CNpredicted-CNcalculated| >0.3.; *c)* removal of all samples with z score ≥2.65 and *d)* removal of all samples with 2.65>z score ≥1.75 and |CNpredicted-CNcalculated| >0.3. (criteria for quality control were adopted from the CopyCaller Software User Guide (PN 4400042B)). Sample sizes before and after quality control are given in Supplementary Table S1a and S1b in File S1.

Allele and carrier frequencies were compared between long-lived cases and younger controls by Fisher's exact test. For the comparison of the different age subgroups, the Armitage trend test was applied. To evaluate whether differences in C4B*Q0 carrier frequency were detected in smokers, we performed a stratified analysis for smokers and non-smokers. For *C4A*, *C4B*, *C4L*, and C4S*Q0 carrier frequencies, we additionally applied a sex-stratified analysis.

Primary endpoint of the initial study was the comparison of the C4B*Q0 carrier frequency between LLI and controls, possibly after stratifying for smoking. Secondary endpoints were the *C4A*, *C4L* and C4S*Q0 carrier frequency differences between cases and controls and the comparison along three to five age subgroup by a trend analysis. For the replication sample, the C4L*Q0 carrier frequency comparison between LLI and controls served as primary endpoint while the trend analysis over four age subgroups again was the secondary endpoint.

The data of the initial and replication sample for the analysis of C4L*Q0 carrier frequency were combined by mega analysis: We performed a logistic regression with case-control status as outcome and C4L*Q0 carrier status and study (initial or replication) as influential variables.

Sample size and power calculations were performed with the statistics program ‘BiAS. für Windows’ version 8.03 (http://www.bias-online.de/). For the other statistical calculations the statistics program R was used [44].

### Registration of smoking habits

Smoking behavior was registered at study entry. Due to missing or indistinct information of smoking habits altogether 490 study participants needed to be excluded from the stratified analysis for smoking. A reported smoking status was available for 672 individuals of the LLI and for 503 controls. Current smokers and recent ex-smokers (quit <3 years ago) were combined as ‘smokers’ and compared to ‘non-smokers’ (never smokers and quit ≥3 years ago). Only cigarette smokers were considered in the analysis. More information about smoking is given in Supplementary Table S1a in File S1.

## Results

The power of our study for replicating the difference in C4B*Q0 carrier frequency between the elderly and younger controls, as seen in Hungarians and Icelandic people [36], [37], [45], was calculated to be 100% for the effects in each of the three studies.

**Figure 1:**
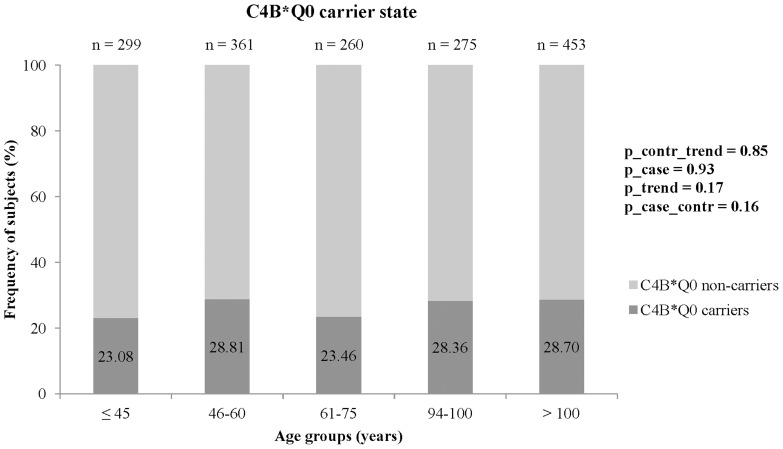
Frequencies of C4B*Q0 carriers (with zero or one *C4B* gene in the diploid genome) in healthy German individuals of different age-groups. p_case_contr: p value for the comparison of C4B*Q0 carrier frequencies between long-lived cases and younger controls (Fisher's exact test; primary endpoint). p_contr_trend: p value for trend test of C4B*Q0 carrier frequencies in the three control subgroups (Armitage trend test; secondary endpoint). p_case: p value for the comparison of C4B*Q0 frequencies in the two case subgroups (Fisher's exact test; secondary endpoint). p_trend: p value for trend test of C4B*Q0 frequencies in all five age groups (Armitage trend test; secondary endpoint).

**Figure 2:**
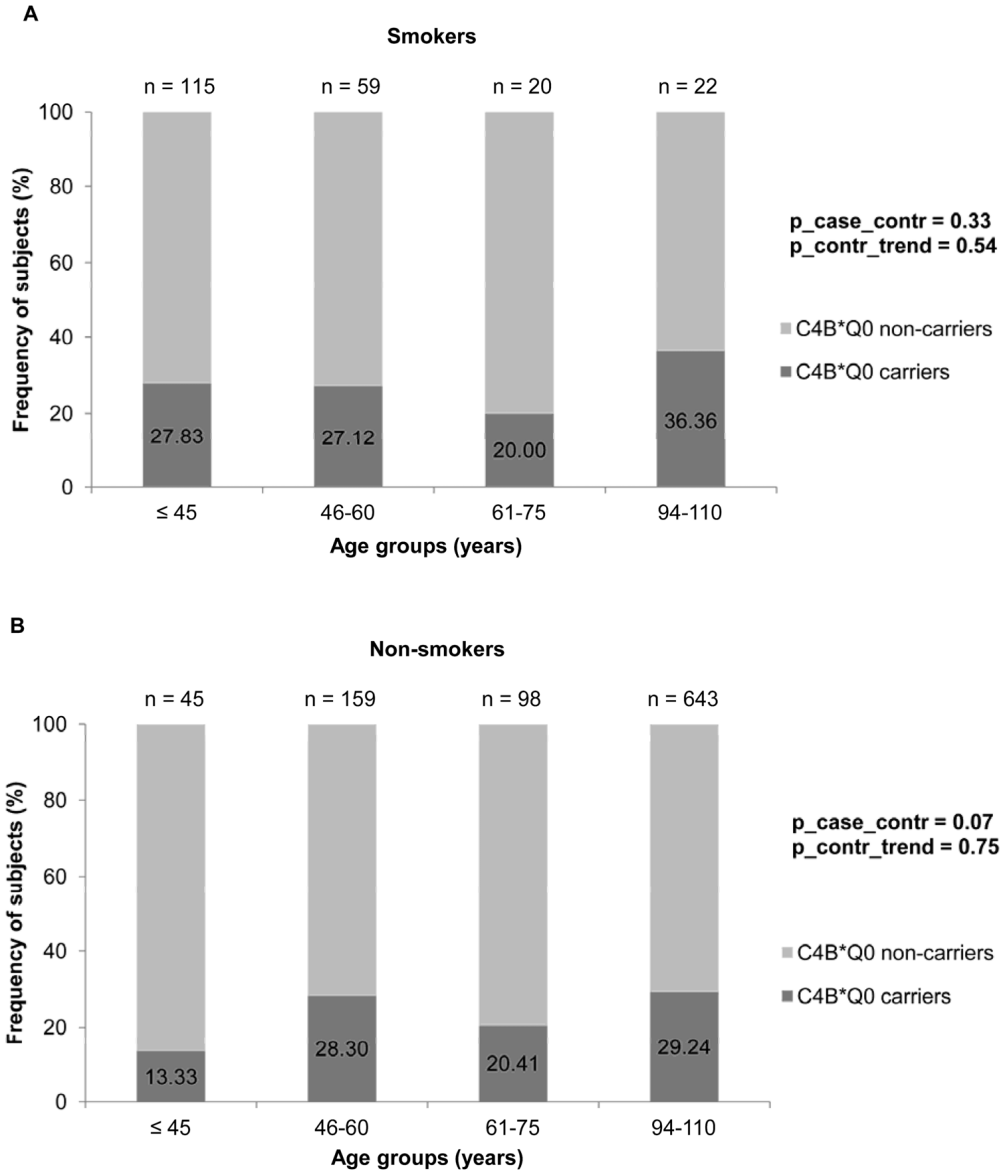
Frequencies of C4B*Q0 carriers in healthy German individuals of different age-groups stratified for (a) smokers (current smokers and quitters <3 years) and (b) non-smokers (never smokers and quitters for ≥3 years). For abbreviations see legend to Figure 1. Due to the small number of centenarians in the replication sample we did not subdivide the case sample into a nonagenarian and centenarian subgroup.

The frequencies for *C4B* copy numbers are given in Table 1 (for *C4A*, *C4S* and *C4L* see Supplementary Table S2 in File S1). They were similar to those previously described for populations of European descent [17], [37]. No difference in frequency distribution was found between long-lived cases and younger controls (p = 0.32). For the primary endpoint C4B*Q0 carrier state (zero or one *C4B* gene in the diploid genome), no difference was found between LLI and the whole group of younger controls (carrier frequency LLI: 28.57%, carrier frequency controls: 25.43%, p = 0.16; Table 2). Due to the wide age-range from 19 to 110 years, the study sample was additionally divided into five different subgroups (secondary endpoint analysis) (≤45 years, 46–60 years, 61–75 years, 94–100 years and >100 years). However, no increasing or decreasing trend in C4B*Q0 carrier frequency was observed for the five age subgroups (p value for trend in the control subgroups: 0.85, p value for the comparison of the two long-lived subgroups: 0.93, p value for trend over all five age subgroups: 0.17) (Table 2). The frequencies varied from 23.08% to 28.81% between different age groups without a trend for age (Figure 1). Since genetic variation may potentially influence the longevity phenotype in men and women differently [46], [47], we also performed a sex-stratified analysis. Again, no significant differences between age groups were detected (data not shown). To evaluate whether differences in C4B*Q0 carrier frequencies would be evident in smokers (as previously described in the study of Szilagyi et al. 2008), we performed a stratified analysis for smokers and non-smokers, but no significant differences were observed (Figure 2). Furthermore, we changed our age grouping such that it was concordant with that of Kramer et al. 1989 [36] and Arason et al. 2003 [37] (Supplementary Table S3 in File S1). Still, no significant difference was detected.

**Table 1:** Frequencies of *C4B* copy numbers (absolute numbers are in parentheses).

	number of *C4B* genes	0	1	2	3	4	5	6	7	p value
German sample	cases (LLI); n = 728	2.34% (17)	26.24% (191)	65.38% (476)	5.49% (40)	0.27% (2)	0.14% (1)	0% (0)	0.14% (1)	
	controls; n = 920	2.28% (21)	23.15% (213)	70.00% (644)	4.35% (40)	0.22% (2)	0% (0)	0% (0)	0% (0)	0.32
European Americans*	females; n = 385	2.3% (9)	25.7% (99)	64.9% (250)	6.8% (26)	0.3% (1)**				
	males; n = 128	3.9% (5)	30.5% (39)	58.6% (75)	7.0% (9)	0% (0)**				

p value: p value for the comparison of allele frequencies between long-lived cases and younger controls (Fisher's exact test).

* adapted from [17]; European American female and male controls.

** number of *C4B* genes ≥4.

**Table 2:** Results of the comparison of Q0 carrier state for the *C4B* gene (absolute numbers are in parentheses).

age groups	≤45 y; n = 299	46–60 y; n = 361	61–75 y; n = 260	94–100 y; n = 275	>100 y; n = 453	p value
C4B*Q0 carriers	23.08% (69)	28.81% (104)	23.46% (61)	28.36 (78)	28.70% (130)	p_contr_trend = 0.85; p_case = 0.93; p_trend = 0.17
	25.43% (234; ≤45–75 y)			28.57% (208; 94–>100 y)		p_case_contr = 0.16

p_contr_trend: p value for trend test of C4B*Q0 frequencies in the three control subgroups (Armitage trend test; secondary endpoint).

p_case: p value for the comparison of C4B*Q0 frequencies in the two case subgroups (Fisher's exact test; secondary endpoint).

p_trend: p value for trend test of C4B*Q0 frequencies in all five age groups (Armitage trend test; secondary endpoint).

p_case_contr: p value for the comparison of C4B*Q0 frequencies between long-lived cases (whole sample) and all younger controls (Fisher's exact test; primary endpoint).

n  =  number; y  =  years.

The investigation of *C4A* and *C4S* copy numbers did not yield a significant difference between age groups (data not shown). In the *C4L* analysis, however, C4L*Q0 demonstrated a different carrier frequency between long-lived cases and younger controls (cases 5.08%, controls 9.12%; p = 0.003, p value for trend in the control subgroups: 0.52, p value for the comparison of the two case subgroups: 0.85, p value for trend over all five age subgroups: 0.004) (Table 3 and Supplementary Table S4 in File S1). The difference between LLI and controls did not depend on gender or smoking status (data not shown). We investigated an additional sample of 714 German LLI and 890 younger controls, which had a power of 86% to replicate our significant results for C4L*Q0. Our primary endpoint analysis revealed a decrease in the C4L*Q0 carrier frequency with age (cases 7.84%, controls 10.00%). Those differences were not statistically significant (p = 0.14), but a significant trend was detected in our secondary endpoint analysis, when the sample was divided into four different age subgroups (≤45 years, 46–60 years, 61–79 years and >91 years, p = 0.03) (Table 4 and Supplementary Table S5 in File S1). When the case-control groups were defined with the same age ranges as the initial screening sample (94–108 and 61–75 years), only marginally different results were obtained (see Supplementary Table S6 in File S1). An additional analysis for males and females separately revealed similar results (data not shown). When the data of the initial and replication sample were combined as a mega analysis (a meta analysis using the original data sets), no significant influence of study (initial or replication) or interaction with C4L*Q0 carrier status was found. However, C4L*Q0 carrier frequency was significantly different between the combined cases and controls (p = 0.002).

**Table 3:** Results of the comparison of Q0 carrier state for the *C4L* gene (absolute numbers are in parentheses).

age groups	≤45 y; n = 294	46–60 y; n = 351	61–75 y; n = 254	94–100 y; n = 233	>100 y; n = 416	p value
C4L*Q0 carriers	9.86% (29)	9.12% (32)	8.27% (21)	4.72 (11)	5.29% (22)	p_contr_trend = 0.52; p_case = 0.85; p_trend = 0.004
	9.12% (82; ≤45–75 y)			5.08% (33; 94–>100 y)		p_case_contr = 0.003

For abbreviations see legend to Table 2.

**Table 4:** Replication study in additional German sample: Results of the comparison of Q0 carrier state for the *C4L* gene (absolute numbers are in parentheses).

age groups	≤45 y; n = 344	46–60 y; n = 248	61–79 y; n = 298	91–108 y; n = 714*	p value
*C4L* * *Q0* carriers	11.92% (41)	10.08% (25)	7.72% (23)	7.84% (56)	p_contr_trend = 0.08; p_trend = 0.03
	10.00% (89; ≤45–79 y)				p_case_contr = .14

For abbreviations see legend to Table 2.

p_trend: p value for trend test of C4B*Q0 frequencies in all four age groups (Armitage trend test; secondary endpoint).

* Due to the small number of centenarians in the replication sample we did not subdivide the case sample into a nonagenarian and centenarian subgroup.

## Discussion

The *C4* gene size (*C4S* or *C4L*) or the copy numbers of total *C4, C4A*, *C4B* genes may influence disease susceptibilities or the immune response [15], [16], [18], [27], [28]. As carriers of unfavorable genetic variants are affected by higher mortality, they are expected to decrease in frequency in population strata of increasing age [8], [9]. Previously, an association between *C4B* copy number and life span was reported for Hungarians [36], [45] showing a decrease of the C4B*Q0 carrier frequency with age from 32% to 10% [45]. This observation indicated that individuals with a low *C4B* copy number could be selected out from the population due to their increased disease mortality [15]. A replication experiment in an independent Icelandic sample also showed a low C4B*Q0 carrier frequency of 5% in the elderly [37]. However, in an Icelandic follow-up study [30], a significant decrease with age was detected for smokers only. In other samples from Italy, no statistically significant differences in C4B*Q0 allele frequencies were observed [40]. The last four studies had a power above 99% to replicate the original C4B*Q0 association [36], irrespective of any interacting effect of smoking. It has to be taken into account, however, that overestimation of effect sizes is a common phenomenon in discovery samples, which may cause subsequent studies to be underpowered [48].

The previous inconsistent association observations prompted us to investigate the role of *C4* copy number variation in our large German longevity sample, taking into consideration the smoking status. No significant frequency differences for *C4A*, *C4B* and *C4S* alleles and no influence of smoking status or gender were detected. In the German sample, the C4B*Q0 carrier frequency for smokers and non-smokers varied from 13.33% to 36.36% for the different age groups without any correlation with age (Figure 2).

For a better comparison with the previous studies, we also subdivided our cases and controls into five age subgroups. Again, no consistent trend for increasing age and no threshold for lower or higher C4B*Q0 carrier frequencies was seen. In particular, the frequencies between nonagenarians and centenarians were very similar.

Considering that our study had a power of 100% to replicate the previous findings, our results suggest that in Germans, the C4B*Q0 copy number plays no role in the ability to reach old age and that smoking does not influence the C4B*Q0 status across age groups as an interacting variable. However, one has to consider that smoking behaviour in prevalence and quality is known to have changed over time during the 20th century [49], [50] and can also vary between different populations. This may result in “smoking” meaning different things in different age groups and populations leading to inconsistent findings. It has also to be taken into account that life expectancy slightly differs between the three investigated populations (Hungary 71.4 years, Germany 77.4 years, Iceland 79.4 years, life expectancies averaged over both sexes according to the U.S. Census Bureau's International Data Base; www.census.gov/). Besides, the negative findings in Germans may be due to population-specific effects, since longevity in different populations is likely to be influenced by varying sets of interacting genetic and environmental factors [51]. With respect to the current investigation, all populations analyzed so far have been of European ancestry and are similar in their *C4* gene variation [20], [22].

Moreover, our German sample was more extensive and the 700 German LLI much older (94–110 years) compared to the Hungarian (60–90 years) and Icelandic (60–93 years) old-age groups. The elderly Hungarians and Icelanders, which showed a significant decrease in the C4B*Q0 allele frequency, just comprised 482, 131, 58 and 25 individuals, respectively [30], [36], [37], [45]. These groups might therefore be prone to a large sampling variance or could represent biased samples that do not yield adequately precise estimates for the intended purposes [52]. This assumption is supported by the observation that deviations in C4B*Q0 frequencies – but without a decreasing trend with age – were seen for those age groups of German smokers and non-smokers that included only very few individuals (smokers: 61–75 years, n number of centenarians in the 20, 20.00%; 94–110 years, n number of centenarians in the 22, 36.36%; Figure 2a), (non-smokers: ≤45 years, n number of centenarians in the 45, 13.33%; 61–75 years, n number of centenarians in the 98, 20.41%; Figure 2b). Overall, our data confirm the negative association result of the Italian centenarian study [40] and suggest that C4B*Q0 copy number does not influence survival into old age.

For *C4L*, independent of gender or smoking status, a significantly lower C4L*Q0 carrier frequency was observed for the long-lived cases compared to younger controls, showing only marginal frequency differences between the three control subgroups on the one hand and between nonagenarians and centenarians on the other. However, our significant case-control association result for the C4L*Q0 allele as primary endpoint could not be replicated in a second German longevity sample, although the same trend towards a decrease in the frequency of C4L*Q0 carriers with age was observed together with a significant trend over four different age groups. Interestingly, the age groups ≤45 years and between 46 and 60 years show a similar C4L*Q0 carrier frequency of around 10 to 12% in contrast to a frequency of around 7 to 8% in the age groups 61 to 79 years and above 91 years (due to the small number of centenarians in the replication sample we did not divide the case sample into a nonagenarian and centenarian subgroup). For the C4L*Q0 replication experiment, a very large analysis population was investigated (1604 individuals), which had a power of 86% to replicate our initial finding. However, it should be mentioned that very often the detected effect size in the first pilot study is considerably overestimated and therefore needs to be adjusted in subsequent replications [48]. Regarding the observed frequency difference for C4L*Q0 carriers in the replication sample (elderly 7.84%; young 10.00%), a sample size of 2825 old-aged cases (with a case-control ratio of 1) would be required for 80% power. Hence, for validation it may be necessary to enlarge sample sizes in future studies or to perform meta-analyses across different longevity populations [53].

To the best of our knowledge, little is known about the association of *C4L* or C4L*Q0 with disease or other phenotypes. The *C4L* variant was shown to be associated with less effective gene transcription compared to the *C4S* variant, raising the possibility of a negative epistatic effect on the expression of C4 proteins [18], [22], [54]. Consequently, the function of the classical complement pathway may affect, via a changed equilibrium or reduction of C4 gene products, complement-dependent pathological processes and hence might influence immunity and lifespan [45]. As the presence of long *C4* genes correlates with lower plasma C4 protein concentrations [18], [22], we hypothesize that *C4L* deficiency in our LLI sample could result in a higher and more balanced C4 protein expression leading to improved health and prolonged life span.

With altogether more than 3000 individuals investigated our study presents the largest *C4* copy number investigation in human longevity research to-date. Thus, our analysis results offer a valuable reference point for further genetic studies and help provide a balanced view of the obtained research evidences.

## Supporting Information

File S1 **Combined file of supporting tables.** Supplementary Table S1a: Characteristics of samples. Supplementary Table S1b: Quality control of *C4A, C4B, C4S* and *C4L.* Supplementary Table S2: Frequencies of *C4A, C4S* and *C4L* copy numbers. Supplementary Table S3: Results of the comparison of Q0 carrier state for the *C4B* gene with age groups as in Kramer et al. 1989 and Arason et al. 2003. Supplementary Table S4: Results of the comparison of Q0 carrier state for the *C4L* gene (carrier and non-carrier information). Supplementary Table S5: Results Replication study in additional German sample: Results of the comparison of Q0 carrier state for the *C4L* gene (carrier and non-carrier information). Supplementary Table S6: Replication study in additional German sample: Results of the comparison of Q0 carrier state for the *C4L* gene with age groups as in the initial study.
(PDF)

Data S1 **Data of copy number calculations performed using the CopyCallerTM Software (Applied Biosystems, Foster City, California, USA) for** ***C4A, C4B, C4S, C4L*** **and** ***C4L*** **Replication.**
(XLSX)
