# Supplementary material for: Investigation of Complement Component C4 Copy Number Variation in Human Longevity
Source: PLoS One. 2014 Jan 22;9(1):e86188. doi: 10.1371/journal.pone.0086188 (PMC3899116; doi:10.1371/journal.pone.0086188)
Supplement: File S1 — Combined file of supporting tables. Supplementary Table S1a: Characteristics of samples. Supplementary Table S1b: Quality control of C4A, C4B, C4S and C4L. Supplementary Table S2: Frequencies of C4A, C4S and C4L copy numbers. Supplementary Table S3: Results of the comparison of Q0 carrier state for the C4B gene with age groups as in Kramer et al. 1989 and Arason et al. 2003. Supplementary Table S4: Results of the comparison of Q0 carrier state for the C4L gene (carrier and non-carrier information). Supplementary Table S5: Results Replication study in additional German sample: Results of the comparison of Q0 carrier state for the C4L gene (carrier and non-carrier information). Supplementary Table S6: Replication study in additional German sample: Results of the comparison of Q0 carrier state for the C4L gene with age groups as in the initial study. (PDF) [file pone.0086188.s001.pdf]

Investigation of complement component *C4* copy number variation in human longevity

ST – Supplementary Tables

Supplementary Table S1a. Characteristics of samples

		Cases	Controls
C4A	**Numbers before quality control**	784	993
	**Numbers after quality control**	724	923
	**Age**<br>Median<br>IQR<br>Min – max	 101<br>97-101<br>94-110	 53<br>41-62<br>19-75
	**Sex** (male:female)	363:361	491:432
	**Smoking***<br>Smokers<br>Non- smokers<br>No information	 21<br>640<br>63	 195<br>306<br>422
C4B	**Numbers before quality control**	784	993
	**Numbers after quality control**	728	920
	**Age**<br>Median<br>IQR<br>Min – max	 101<br>97-101<br>94-110	 53<br>41-62<br>19-75
	**Sex** (male:female)	366:362	490:430
	**Smoking***<br>Smokers<br>Non-smokers<br>No information	 22<br>643<br>63	 194<br>302<br>424
C4S	**Numbers before quality control**	784	993
	**Numbers after quality control**	736	929
	**Age**<br>Median<br>IQR<br>Min – max	 101<br>97-101<br>94-110	 53<br>41-62<br>19-75
	**Sex** (male:female)	375:361	493:436
	**Smoking***<br>Smokers<br>Non-smokers<br>No information	 22<br>650<br>64	 198<br>305<br>426

C4L	**Numbers before quality control**	784	993
	**Numbers after quality control**	649	899
	**Age**<br>Median<br>IQR<br>Min – max	 101<br>98-101<br>94-110	 53<br>41-62<br>19-75
	**Sex** (male:female)	321:328	476:423
	**Smoking***<br>Smokers<br>Non-smokers<br>No information	 18<br>575<br>56	 191<br>294<br>414
***C4L* replication**	**Numbers before quality control**	823	1202
	**Numbers after quality control**	714	890
	**Age**<br>Median<br>IQR<br>Min – max	 98<br>96-99<br>91-108	 50<br>40-65<br>18-79
	**Sex** (male:female)	59:655	90:800

IQR: interquartile range, *: “smokers” were defined as current smokers and recent ex-smokers (quit <3 years ago), “non-smokers” as never smokers and quit ≥3 years ago

For the *C4L* replication, smoking was not investigated.

Supplementary Table S1b. Quality control of *C4A*, *C4B*, *C4S* and *C4L*

	C4A		***C4B***		***C4S***		***C4L***		***C4L* replication**	
	Cases	Controls	Cases	Controls	Cases	Controls	Cases	Controls	Cases	Controls
Before qc	784	993	784	993	784	993	784	993	823	1202
Replicate Number	770	955	771	953	766	947	770	956	822	1133
Confidence	745	937	757	945	755	937	663	903	716	890
Z score ≥ 2.65	741	934	746	929	752	934	663	903	715	890
2.65 > z score ≥ 1.75	738	927	742	924	750	933	663	903	714	890
C4A+C4B≠C4L+C4S	724	923	728	920	736	929	649	899		

Before qc: numbers before quality control

Replicate number: removal of all samples with analyzed replicate number 0, 1 or 2

Confidence: removal of all samples with confidence < 0.95 and |CNpredicted-CNcalculated|> 0.3

Z score ≥ 2.65: removal of all samples with z score ≥ 2.65

2.65 > z score ≥ 1.75: removal of all samples with 2.65 > z score ≥ 1.75 and |CNpredicted-CNcalculated|> 0.3

C4A+C4B≠C4L+C4S: removal of all samples with sum of *C4A* copies plus *C4B* copies not equal to the sum of *C4L* copies plus *C4S* copies (not available for *C4L* replication)

Supplementary Table S2. Frequencies of *C4A*, *C4S* and *C4L* copy numbers (absolute numbers are in parentheses)

	number of genes	0	1	2	3	4	5	6	10	p value
C4A	cases (LLI) n = 724	(5) 0.69%	(118) 16.30%	(426) 58.84%	(156) 21.55%	(19) 2.62%	(0) 0%	(0) 0%	(0) 0%	0.72
	controls n = 923	(10) 1.08%	(169) 18.31%	(517) 56.01%	(204) 22.10%	(22) 2.38%	(1) 0.11%	(0) 0%	(0) 0%	
C4S	cases (LLI) n = 736	(274) 37.23%	(342) 46.47%	(105) 14.27%	(13) 1.77%	(1) 0.14%	(1) 0.14%	(0) 0%	(0) 0%	0.51
	controls n = 929	(317) 34.12%	(437) 47.04%	(158) 17.01%	(13) 1.40%	(3) 0.32%	(1) 0.11%	(0) 0%	(0) 0%	
C4L	cases (LLI) n = 649	(1) 0.15%	(32) 4.93%	(172) 26.50%	(279) 42.99%	(154) 23.73%	(11) 1.69%	(0) 0%	(0) 0%	0.00065
	controls n = 899	(9) 1.00%	(73) 8.12%	(206) 22.91%	(328) 36.48%	(269) 29.92%	(13) 1.45%	(1) 0.11%	(0) 0%	
C4L replication	cases (LLI) n = 714	(10) 1.40%	(46) 6.44%	(176) 24.65%	(273) 38.24%	(184) 25.77%	(14) 1.96%	(10) 1.40%	(1) 0.14%	0.000021
	controls n = 890	(9) 1.01%	(80) 8.99%	(273) 30.67%	(342) 38.43%	(171) 19.21%	(15) 1.69%	(0) 0%	(0) 0%	

p value: p value for the comparison of allele frequencies between long-lived cases and younger controls (Fisher´s exact test)

Supplementary Table S3. Results of the comparison of Q0 carrier state for the *C4B* gene with age groups as in Kramer et al. 1989 and Arason et al. 2003

a. **Age distribution of cases and controls according to Kramer et al. 1989**

	controls ≤53 years n = 463	cases (LLI) 61 - 110 years n = 970	p value
*C4B*Q0* carriers	n = 118 25.49%	n = 265 27.32%	0.48

b. **Age distribution of cases and controls according to Arason et al. 2003**

	controls ≤54 years n = 487	cases (LLI) 55 - 110 years n =1161	p value
*C4B*Q0* carriers	n = 125 25.67%	n = 317 27.30%	0.50

p value: p value for the comparison of *C4B*Q0* frequencies between long-lived cases and younger controls (Fisher´s exact test)

Supplementary Table S4. Results of the comparison of Q0 carrier state for the *C4L* gene (carrier and non-carrier information)

	≤ 45<br>n = 294	46 - 60<br>n = 351	61 - 75<br>n = 254	94 - 100<br>n = 233	> 100<br>n = 416	p value
*C4L*Q0* carriers	n = 29<br>9.86%	n = 32<br>9.12%	n = 21<br>8.27%	n = 11<br>4.72%	n = 22<br>5.29%	p_contr_trend = 0.52<br>p_case = 0.85<br>p_trend = 0.004
	all controls<br>n = 82<br>9.12%			all cases (LLI)<br>n = 33<br>5.08%		p_case_contr = 0.003
*C4L*Q0* non-carriers	n = 265<br>90.14%	n = 319<br>90.88%	n = 233<br>91.73%	n = 222<br>95.28%	n = 394<br>94.71%	
	all controls<br>n = 817<br>90.88%			cases (LLI)<br>n = 616<br>95.92%		

p_case_contr: p value for the comparison of *C4L*Q0* frequencies between long-lived cases and younger controls (Fisher´s exact test; primary endpoint)

p_contr_trend: p value for trend test of *C4L*Q0* frequencies in the three control subgroups (Armitage trend test; secondary endpoint)

p_case: p value for the comparison of *C4L*Q0* frequencies in the two case subgroups (Fisher´s exact test; secondary endpoint)

p_trend: p value for trend test of *C4L*Q0* frequencies in all five age groups (Armitage trend test; secondary endpoint)

Supplementary Table S5. Results Replication study in additional German sample: Results of the comparison of Q0 carrier state for the *C4L* gene (carrier and non-carrier information)

	≤ 45<br>n = 344	46 - 60<br>n = 248	61 - 79<br>n = 298	91 – 108*<br>n = 714		p value
*C4L*Q0* carriers	n = 41<br>11.92%	n = 25<br>10.08%	n = 23<br>7.72%			p_contr_trend = 0.08<br>p_trend = 0.03
	all controls<br>n = 89<br>10.00%			cases (LLI)<br>n = 56<br>7.84%		p_case_contr = 0.14
*C4L*Q0* non-carriers	n = 303<br>88.08%	n = 223<br>89.92%	n = 275<br>92.28%	n = 577<br>92.62%	n = 81<br>89.01%	
	all controls<br>n = 801<br>90.00%			cases (LLI)<br>n = 658<br>92.16%		

***** due to the small number of centenarians in the replication sample we did not subdivide the case sample into a nonagenarian and centenarian subgroup

For abbreviations see legend to Supplementary Table 4.

Supplementary Table S6. Replication study in additional German sample: Results of the comparison of Q0 carrier state for the *C4L* gene with age groups as in the initial study

<table>
<tr><td></td><td>≤ 45
n = 344</td><td>46 - 60
n = 248</td><td>61 - 75
n = 254</td><td>94 - 108*
n = 707</td><td>p value</td></tr>
<tr><td rowspan="2">C4L*Q0
carriers</td><td>n = 41
11.92%</td><td>n = 25
10.08%</td><td>n = 19
7.48%</td><td></td><td>p_contr_trend = 0.08
p_trend=0.03</td></tr>
<tr><td colspan="3">all controls
n = 85
10.05%</td><td>cases (LLI)
n = 56
7.92%</td><td>p_case_contr = 0.16</td></tr>
</table>

For abbreviations see legend to Supplementary Table 4.
